# Carotid disease at age 73 and cognitive change from age 70 to 76 years: A longitudinal cohort study

**DOI:** 10.1177/0271678X16683693

**Published:** 2016-01-01

**Authors:** Joanna M Wardlaw, Michael Allerhand, Elizabeth Eadie, Avril Thomas, Janey Corley, Alison Pattie, Adele Taylor, Susan D Shenkin, Simon Cox, Alan Gow, John M Starr, Ian J Deary

**Affiliations:** 1 Brain Research Imaging Centre, Centre for Clinical Brain Sciences, University of Edinburgh, Edinburgh, UK; 2 Department of Neuroradiology, NHS Lothian, Western General Hospital, Edinburgh, UK; 3 Centre for Cognitive Ageing and Cognitive Epidemiology, University of Edinburgh, Edinburgh, UK; 4 Geriatric Medicine, University of Edinburgh, Royal Infirmary, Edinburgh, UK; 5 Department of Psychology, Heriot-Watt University, Edinburgh, UK

**Keywords:** Carotid stenosis, white matter hyperintensities, cognition, ageing, vascular risk factors

## Abstract

Cognitive decline and carotid artery atheroma are common at older ages. In community-dwelling subjects, we assessed cognition at ages 70, 73 and 76 and carotid Doppler ultrasound at age 73, to determine whether carotid stenosis was related to cognitive decline. We used latent growth curve models to examine associations between four carotid measures (internal carotid artery stenosis, velocity, pulsatility and resistivity indices) and four cognitive ability domains (memory, visuospatial function, crystallised intelligence, processing speed) adjusted for cognitive ability at age 11, current age, gender and vascular risk factors. Amongst 866 participants, carotid stenosis (median 12.96%) was not associated with cognitive abilities at age 70 or cognitive decline from age 70 to 76. Increased ICA pulsatility and resistivity indices were associated with slower processing speed (both *P* < 0.001) and worse visuospatial function (*P* = 0.036, 0.031, respectively) at age 70, and declining crystallised intelligence from ages 70 to 76 (*P* = 0.008, 0.006, respectively). The findings suggest that vascular stiffening, rather than carotid luminal narrowing, adversely influences cognitive ageing and provides a potential target for ameliorating age-related cognitive decline.

## Introduction

Carotid stenosis increases with age, is a major risk factor for embolic stroke,^1^ and may reduce cerebral blood supply. Cognitive decline and dementia also increase with advancing age,^2^ and vascular risk factors and stroke are associated with higher rates of cognitive decline and dementia.^3,4^ White matter hyperintensities (WMH) are a common finding indicative of vascular disease on neuroimaging in older people, are associated with reduced cerebral blood flow,^5^ cause cognitive decline and increase the risk of dementia and stroke.^6^

Carotid stenosis induced by placing wires around the internal carotid arteries (ICA) is used as a model of ageing-related cognitive impairment (Holland et al., 2015).^7^ However, in humans, the association between carotid stenosis and cognition is less clear.^8,9^ In several studies (total n = 12,887), the presence of carotid atheroma as indicated by plaque area or intima-media thickening (IMT), but not luminal narrowing was associated with declining cognitive function or dementia.^10–13^ The Framingham study (n = 1975) found variable associations between carotid IMT or stenosis > 50% and cognitive function or WMH.^14^ However, carotid endarterectomy to remove tight stenosis did not influence cognitive decline five years later.^15^ Other studies (total n = 1300)^16,17^ found the cross-sectional association between carotid IMT or stenosis and WMH was due to shared co-association with age and vascular risk factors. Several studies found that increased systemic vascular or ICA stiffness, as indicated by raised pulse pressure or carotid pulsatility or resistivity indices, was associated with declining cognitive function^18,19^ and/or WMH.^19,20^ No studies examined trajectories of different cognitive domains and their associations with markers of carotid atheroma and stiffness while correcting for prior cognitive ability, a major determinant of cognitive ability at older ages.^21^

The present analysis aimed to determine if carotid stenosis is associated independently with performance level and/or change in key cognitive domains between ages 70 and 76 in a large, narrow-age sample of community-dwelling subjects.

## Methods

### Participants

The Lothian Birth Cohort 1936 is a prospective study of subjects born in 1936, most of whom took a general mental ability test in the Scottish Mental Survey of 1947 (SMS 1947) at age 11. Those living in the Edinburgh area at about age 70 years were recruited into a longitudinal study of ageing. The protocols,^22,23^ cognitive profiles,^24^ vascular risk factors^17,25^ and other relevant analyses^26^ are published.

Cognitive, physical and health assessments were performed at mean ages of 70, 73 and 76 years; carotid Doppler ultrasound (DUS) was performed at age 73. Carotid imaging and all data analyses were blind to all other test results including cognition.

### Ethics and reporting guidelines

The study was approved by the Multi-Centre Research Ethics Committee for Scotland (Wave-1: MREC/01/0/56), the Lothian Research Ethics Committee (Wave-1: LREC/2003/2/29), and the Scotland A Research Ethics Committee (Waves-2 and 3: 07/MRE00/58). Written informed consent was obtained from all participants. The study was conducted according to the Strengthening the Reporting of Observational Studies in Epidemiology (STROBE) Guidelines.

### Health assessments

Health assessments included medical history, physiological measurements, and blood samples (full details in Deary et al.^22^). We recorded self-reported (at interview) hypertension, diabetes, hypercholesterolemia (all either medically diagnosed and/or on relevant drugs), history of ischaemic heart disease (IHD), stroke, peripheral vascular disease, or other circulatory problems (diagnosed by GP or hospital doctor), and smoking (current versus stopped > 1 year ago or never smoked). Blood pressure (average of three sitting and standing measures at each of three waves of testing), plasma haemoglobin A1c (HbA1C) and total serum cholesterol were measured.

### Cognitive functions

Cognitive functions were tested in four domains designed to measure multiple aspects of ageing-relevant cognitive ability: visuospatial ability, processing speed, memory, and crystallized intelligence.^22,27^ Visuospatial ability was measured by Matrix Reasoning and Block Design from the Wechsler Adult Intelligence Scale Third Edition, WAIS-III, and Spatial Span Forward and Spatial Span Backward from the Wechsler Memory Scale Third Edition, WMS-III. Memory was measured by Logical Memory, Verbal Paired Associates, and Digit Span Backwards from the WMS-III. Processing speed was measured by Symbol Search and Digit Symbol from the WAIS-III, Choice Reaction Time and Inspection Time. Crystallized intelligence was measured by the National Adult Reading Test (NART), the Wechsler Test of Adult Reading (WTAR), and Verbal Fluency (C,F,L).^22,27^ We included, as a covariate, participants’ cognitive ability scores on the Moray House Test No. 12 from the SMS1947, obtained when participants were aged 11 years.^22^

### Carotid ultrasound imaging

Carotid ultrasound imaging was performed on a Siemens Antares Premium Colour Doppler scanner (Siemens AG, Erlangen, Germany) with 7.5 MHz variable frequency probe by experienced neurovascular ultrasonographers, all cross checked by a consultant neuroradiologist. We recorded: carotid IMT in the common carotid arteries (CCA) and carotid bulbs bilaterally (mean of three calliper measures from each artery); flow velocities (after at least five minutes rest supine with head on pillow) in the ICA (peak systolic, end diastolic), common carotid artery (CCA), external carotid artery (ECA) and vertebral artery (VA) bilaterally. We determined the maximum stenosis affecting the ICA (or carotid bulb or CCA if located there) using validated calliper measurement^28^ and ICA peak and end diastolic velocities and ICA/CCA peak systolic velocity ratio,^29^ according to the North American Symptomatic Carotid Endarterectomy Trial (NASCET) definition.

### Statistical modelling

To test the assumption that the pattern of missing values due to inter-wave dropout was acceptably random, we explored the potential influence of missing observations upon inferential results by considering the prognostic effect of subsequent dropout on each of the observed variables. For this we divided the study sample into two: those who completed the study, and those who dropped-out at some time after the first measurement at age 70. We tested for differences in participant characteristics between these two groups, using t-tests or Pearson Chi-square as appropriate.

We grouped the cognitive tests into four categories representing: visuospatial ability, processing speed, memory and crystallized ability, as explained previously.^27^ We standardised the cognitive tests and arranged the scale directions so that lower score = lower (worse) cognitive function. We developed measurement models for each of the four cognitive domains separately and tested their longitudinal invariance across three measurement occasions (at ages 70, 73 and 76 years, corrected for age 11 IQ) using the R semTools package. The models were developed in a standard procedure: explore different orders for the indicators, eliminate negative residual variances, free residual covariances according to modification indices to obtain best goodness of fit, then test and assess longitudinal measurement invariance.

We derived measures of carotid disease taking the average of right and left values. We used maximum ICA stenosis (0 to 100%),^28^ the peak systolic ICA velocity, the pulsatility and the resistivity indices calculated from the ICA velocity waveform.^20^ The stenosis percentages and the ICA velocity, pulsatility and resistivity indices, were categorised on an ordinal scale derived from the sample quintiles. The stenosis values were divided into 10% steps (0–9%, 10–19%, etc.) to reduce skewness. The peak ICA velocities were grouped by 10% increments of velocity (cm/s). The pulsatility and resistivity indices were analysed in raw units. We also tested the four measures in their standard deviation units (equivalent to “beta weights” in multiple regression) so their effects were more comparable. Although carotid pulsatility and resistivity were highly correlated, being related by a formula,^20^ we modelled both separately to compare their effects.

**Figure 1.**
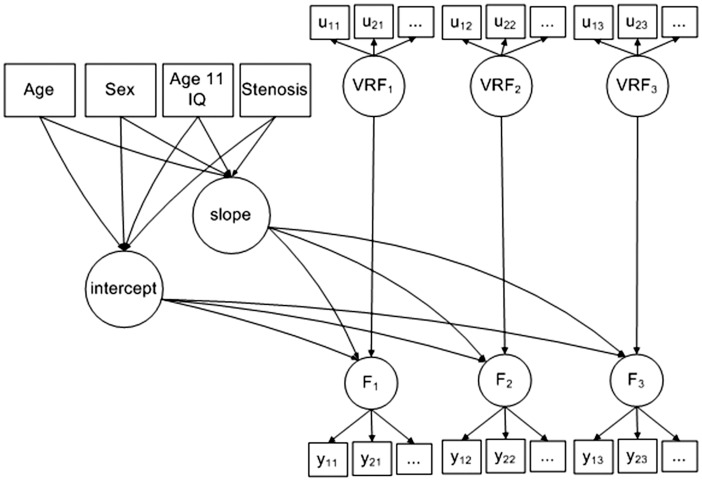
Diagram of the model developed to test the association between cognitive abilities at ages 70, 73 and 76, vascular risk factors and carotid stenosis. Note: The u_ij_ are repeated measurements of vascular risk, (VRF), *i*th variable at *j*th time-point: diastolic blood pressure (average of three tests, sitting), blood cholesterol, blood (hba1c), history of cardiovascular disease, history of high blood pressure, and history of high cholesterol. The y_ij_ are repeated measurements of cognitive function (F). Four kinds of cognitive function were modelled separately: visuospatial processing, (indicated by task performance in matrix reasoning, block design, spatial span forward, and spatial span backward), memory, (indicated by verbal paired associates, logical memory, and digit span backwards), processing speed, (indicated by choice reaction time, inspection time, digit symbol search, and symbol search), and crystallized intelligence, (indicated by NART, WTAR, and verbal fluency). The repeated measurements were made on three occasions of testing at ages 70, 73 and 76, respectively. The measurement time points have average interval 3.3 years, (SD = 0.5 years). Factors ‘intercept’ and ‘slope’ represent the baseline level at age 70 and the rate of change of the cognitive ability factor (F) from age 70 to 76. Age at baseline, sex, age 11 IQ, and stenosis are time-invariant covariates with effects on the intercept and slope of the linear trajectory of cognition over time. Age at baseline was centred on 70 years. Sex was coded 0 for female and 1 for male. Age 11 IQ was centred on 100 and scaled for standard deviation of 15. Four kinds of stenosis measure were considered in turn: percentage stenosis, velocity, pulsatility, and resistivity. The percentage stenosis measured as the average of left and right carotid stenosis, and the resulting percentages were categorised on an ordinal scale cut in 10% stenosis groups.

We developed latent growth models (Figure 1) for each of the four cognitive domains. We included the four carotid measures (and age at baseline and sex as covariates) in each model to test the association between incremental increases in stenosis, ICA velocity, pulsatility and resistivity indices, on the intercept (i.e. association with cognitive abilities at age 70) and slope of the trajectory of cognitive change (i.e. change in cognitive abilities between ages 70 and 76). We used all available data and did not impute any missing variables. Age was centred on 70 years. Sex was centred on female. Details of model loadings are given in Supplementary Tables 1 (intercept) and 2 (slope). Goodness of fit was assessed using Chi-square, the RMSEA, and CFI, provided by structural equation software under maximum likelihood estimation. The model controlled time-varying fluctuations in vascular risk by including a vascular risk factor latent variable (VRF) at each time-point. Vascular risk was measured by diastolic blood pressure, blood cholesterol, blood HBA1c, history of cardiovascular disease, hypertension, and hypercholesterolaemia as previously^17^ which developed a well-fitting VRF latent trait (RMSEA = 0.02, CFI = 0.99), i.e. a measurement model using structural equation modelling. This allowed us to compare the association between stenosis and cognition with and without controlling for VRF. Including VRF also allowed us to test its effect as a time-varying predictor upon fluctuation in cognitive function between ages 70 and 76. Finally, we tested associations between carotid stenosis, ICA velocity, pulsatility and resistivity indices per standard deviation increase (rather than by incremental increases) and cognition at age 70 (intercept) and change in cognition between age 70 and 76 (slope). We did not adjust for multiple comparisons because in any one analysis, there were relatively few comparisons (we assessed each of the four carotid parameters separately, and used latent variables rather than raw individual risk factors).

## Results

There were 1091 participants of mean age 69.53 (SD ± 0.83) at wave-1 (49.8% were female), 866 participants of mean age 72.49 (SD ± 0.71) at wave-2 and 697 participants of mean age 76.25 (SD ± 0.68) at wave 3. The median stenosis obtained in 820 participants at wave 2 was 10.0% (mean 12.96%), of whom 1.7% had > 50% stenosis, and 0.12% had > 75% stenosis.

Table 1 gives the cognitive test results, vascular risk factor and imaging findings at each wave. The proportion of participants with hypertension, diabetes, hypercholesterolaemia and cardiovascular disease increased, and of current smokers fell, between waves-1 and -3. This reflects that the participants who provided data at all three waves (‘completers’) differed significantly from those who did not (‘dropouts’; Table 2). Those who provided data at all three waves had higher IQ at age 11 (101.54 vs. 97.3, *P* < 0.001), performed better on all current tests of cognition in all domains (all *P* < 0.001), were less likely to be current smokers (7% vs. 19%, *P* < 0.001) or to have hypercholesterolaemia (33% vs. 44%, *P* = 0.015). However, they were no different in terms of carotid parameters, proportion with diabetes, hypertension or cardiovascular disease.

**Table 1.** Characteristics of participants at ages 70, 73 and 76 years.

	Age 70	Age 73	*P* ^a^	Age 76	P^b^
N	1091	866		697	
Sex
Female	543 (49.8)				
Male	548 (50.2)				
Age	69.53 (0.83)	72.49 (0.71)	<.001	76.25 (0.68)	<.001
IQ age 11	100 (14.99)				
Crystallised:	
Wechsler test of adult reading	41.02 (7.17)	41.01 (6.97)	0.973	41.09 (7.03)	0.819
National adult reading test	34.48 (8.15)	34.38 (8.18)	0.783	35 (8.04)	0.132
Verbal fluency	42.42 (12.54)	43.18 (12.94)	0.19	42.9 (12.76)	0.666
Memory:
Logical memory	44.07 (10.43)	45.56 (10.39)	0.002	45.75 (10.94)	0.723
Verbal paired associates	20.23 (7.41)	20.8 (7.68)	0.104	20.13 (7.76)	0.096
Digit span backwards	7.73 (2.26)	7.81 (2.29)	0.445	7.77 (2.37)	0.701
Speed:
Inspection time	112.14 (11)	111.22 (11.79)	0.084	110.17 (12.53)	0.099
Choice reaction time	0.64 (0.09)	0.65 (0.09)	0.075	0.68 (0.1)	<.001
Digit symbol	56.6 (12.93)	56.4 (12.31)	0.729	53.81 (12.93)	<.001
Symbol search	24.71 (6.39)	24.61 (6.18)	0.736	24.6 (6.46)	0.988
Visuo-spatial:
Spatial span forward	7.68 (1.64)	7.63 (1.66)	0.54	7.57 (1.63)	0.44
Block design	33.79 (10.32)	33.64 (10.08)	0.745	32.18 (9.95)	0.004
Matrix reasoning	13.49 (5.13)	13.17 (4.96)	0.165	13.04 (4.91)	0.59
Spatial span backward	7.04 (1.74)	7.06 (1.61)	0.769	7.05 (1.59)	0.858
Carotid:
Stenosis (%)		12.96 (N = 820)			
Velocity (m/s)		32.26 (N = 817)			
Pulsatility index		1.28 (N = 817)			
Resistivity index		0.68 (N = 817)			
Diabetes:
Yes	91 (8.3)	95 (11)	<.001	82 (11.8))	<.001
Smoking:
Current	125 (11.5)	73 (8.4)	<.001	44 (6.3)	<.001
Ex	465 (42.6)	378 (43.6)		293 (42.1)	
Never	501 (45.9)	415 (47.9)		359 (51.6)	
Systolic BP:	149.78 (19.2)	148.81 (18.95)	0.265	148.31 (19.56)	0.615
Diastolic BP:	81.35 (10.31)	78.07 (9.86)	<.001	78.97 (10.47)	0.083
Cholesterol (total):	5.45 (1.16)	5.15 (1.15)	<.001	5 (1.2)	0.012
HbA1C	5.93 (0.77)	5.75 (0.66)	<.001	5.85 (0.63)	0.006
CVD:
Yes	268 (24.6)	250 (28.9)	<.001	236 (33.9)	<.001
Hypertension:
Yes	433 (39.7)	425 (49.1)	<.001	378 (54.3)	<.001
Hypercholesterolaemia:
Yes	386 (35.4)	356 (41.1)	<.001	328 (47.3)	<.001

a *P*-value reflects significance of difference between ages 70 and 73

b *P*-value reflects significance of difference between ages 73 and 76. Stenosis is mean of left and right.

Note: Values are mean(SD) or N(%) unless otherwise stated.

**Table 2.** Differences between those providing data at all three waves (‘completer’) and those who provided data at only two or one wave (‘dropout’).

	Completer	Dropout	*P*
Age	69.5 (697)	69.59 (394)	0.091
Sex			
Female	48 (337)	52 (206)	
Male	52 (360)	48 (188)	0.212
IQ age 11	101.54 (655)	97.3 (373)	<.001
Crystallised
Wechsler test of adult reading	41.59 (694)	40.03 (395)	<.001
National adult reading test	35.1 (695)	33.4 (394)	<.001
Verbal fluency	43.24 (695)	40.97 (392)	0.003
Memory
Logical memory	45.44 (691)	41.7 (397)	<.001
Verbal paired associates	21.16 (655)	18.71 (404)	<.001
Digit span backwards	7.93 (695)	7.4 (395)	<.001
Speed
Inspection time	113.27 (644)	110.3 (397)	<.001
Choice reaction time	0.63 (685)	0.66 (399)	<.001
Digit symbol	58.45 (684)	53.44 (402)	<.001
Symbol search	25.42 (687)	23.47 (399)	<.001
Visuo-spatial
Spatial span forward	7.79 (689)	7.49 (397)	0.004
Block design	35.09 (690)	31.51 (395)	<.001
Matrix reasoning	14.1 (688)	12.45 (398)	<.001
Spatial span backward	7.2 (687)	6.76 (397)	<.001
Carotid
Stenosis	12.6 (673)	14.6 (147)	0.123
Velocity	32.28 (672)	32.16 (145)	0.919
Pulsatility	1.27 (672)	1.31 (145)	0.176
Resistivity	0.68 (672)	0.69 (145)	0.774
Diabetes
Yes	7 (51)	10 (40)	0.104
Smoking
Current	7 (50)	19 (75)	
Ex	43 (297)	43 (168)	
Never	50 (349)	38 (152)	<.001
SBP	148.92 (696)	151.3 (392)	0.054
DBP	81.09 (696)	81.81 (392)	0.286
Cholesterol	5.45 (620)	5.46 (434)	0.836
HbA1C	5.93 (491)	5.93 (570)	0.95
CVD
Yes	23 (161)	27 (107)	0.145
Hypertension
Yes	38 (262)	43 (171)	0.067
Hypercholesterolaemia
Yes	33 (227)	40 (159)	0.015

Note: The number of subjects in each group is given in parentheses.

At age 70 (i.e. intercept), older age at wave-1 testing (mean 69.53, SD ± 0.83) was associated with significantly worse performance in all four cognitive domains (estimates − 0.134 to − 0.214, all *P* < 0.001). Lower age 11 IQ was also associated with significantly worse performance in all four cognitive domains (estimates 0.035 to 0.047, all *P* < 0.001); in other words, a 10 points higher IQ at age 11 was associated with 0.47 × SD higher score on crystallised, and about a 0.36 × SD higher score on memory, speed and visuospatial cognitive tests at age 70. Being male (vs. female) was associated only with memory (positively) and visuospatial function (negatively), both *P* < 0.001, Table 3.

**Table 3.** Covariate effects on the intercept (age 70) and slope (age 70–76).

		Intercept age 70			Slope (change age 70–76)		
Covariate	Cognitive function	Est	SE	*P*	Est	SE	*P*
Age at baseline	Crystallized	−0.134	0.027	<0.001	0.247	0.102	0.015
	Memory	−0.186	0.039	<0.001	1.092	0.109	<0.001
	Speed	−0.214	0.035	<0.001	0.114	0.088	0.197
	Visuospatial	−0.187	0.035	<0.001	1.004	0.125	<0.001
Sex	Crystallized	0.022	0.045	0.617	0.366	0.167	0.029
	Memory	0.254	0.065	<0.001	0.344	0.399	0.389
	Speed	0.013	0.060	0.826	0.220	0.148	0.137
	Visuospatial	−0.535	0.056	<0.001	0.477	0.353	0.177
IQ at age 11	Crystallized	0.047	0.001	<0.001	0.002	0.004	0.591
	Memory	0.036	0.002	<0.001	-0.007	0.014	0.618
	Speed	0.035	0.002	<0.001	-0.003	0.005	0.562
	Visuospatial	0.037	0.002	<0.001	-0.029	0.011	0.008

Note: Covariates are centred close to their respective means. Age at baseline is centred on 70 years. Sex is coded 0 = female, 1 = male. Age 11 IQ is centred on 100 and is in units of its standard deviation.^16^ The intercept represents the expected level of cognitive function when all covariates are at their centred values, (age 70, female, average IQ at age 11). Each covariate effect represents additive change in the intercept per unit increase in the covariate. Change in the intercept is in units of the standard deviation of its marker variable at wave1, i.e. standardised co-efficients. The slope represents the rate of change in cognitive function, in units of the standard deviation of its marker variable at wave1 per unit time of 3.3 years, (the average wave interval). Each covariate effect represents additive change in the slope per unit increase in the covariate. A positive effect indicates a less negative slope.

Between age 70 and 76, all four cognitive domains declined their rate of decline being between 0.02 and 0.1 standard deviations per three-year interval (all *P* < 0.01, Supplementary Table 2). Considering the slope of cognitive decline between ages 70 and 76 (Table 3), subjects who were older at wave-1 had significantly slower decline in visuospatial function and memory (estimates 1.092 and 1.004, respectively, both *P* < 0.001) with a weak effect on crystallised intelligence (estimate 0.247, *P* < 0.015), but not processing speed. Being male (vs. female) was weakly associated with slower decline in crystallised intelligence (estimate 0.366, *P* = 0.029) but not the other cognitive domains. Higher age 11 IQ was associated with faster decline in visuospatial function but not other cognitive domains (estimate − 0.029, *P* = 0.008, Table 3).

There was no association between age 11 IQ and carotid parameters (Supplementary Table 3). In the eighth decade, neither increasing degrees of carotid stenosis, whether expressed in 10% increments (Table 4) or by standard deviation increases (Supplementary Table 4), nor ICA velocities were associated with either cognition at age 70 or change in cognition between ages 70 and 76, without or with vascular risk factor adjustment. At age 70, increasing pulsatility and resistivity indices were both associated with poorer processing speed (e.g. pulsatility index estimate −0.149, *P* < 0.001; resistivity index − 0.148, *P* = 0.034) and more weakly with visuospatial function (e.g. pulsatility index, estimate − 0.079, *P* = 0.033; resistivity index, estimate − 0.082, *P* = 0.033). Controlling for VRF made little difference to the strength of these associations (Table 4).

**Table 4.** Carotid parameters and cognition aged 70 (intercept) and change between age 70 and 76 (slope).

			Intercept age 70			Slope (change age 70–76)		
	Cognitive function	VRF controlled	Est	SE	*P*	Est	SE	*P*
Stenosis	Crystallized	No	0.007	0.021	0.728	−0.003	0.056	0.959
		Yes	0.011	0.022	0.621	−0.031	0.202	0.877
	Memory	No	0.017	0.030	0.569	−0.297	0.159	0.062
		Yes	−0.008	0.031	0.799	−0.238	0.187	0.203
	Speed	No	−0.044	0.028	0.116	−0.118	0.062	0.058
		Yes	−0.031	0.029	0.273	−0.114	0.066	0.083
	Visuospatial	No	−0.023	0.027	0.396	0.020	0.157	0.897
		Yes	−0.017	0.029	0.556	0.050	0.154	0.744
Carotid velocity	Crystallized	No	0.026	0.025	0.292	−0.040	0.068	0.563
		Yes	0.029	0.026	0.263	−0.185	0.242	0.446
	Memory	No	0.038	0.037	0.305	−0.065	0.224	0.773
		Yes	0.032	0.037	0.394	−0.083	0.252	0.741
	Speed	No	0.025	0.034	0.463	−0.085	0.074	0.251
		Yes	0.026	0.034	0.455	−0.109	0.076	0.149
	Visuospatial	No	0.034	0.033	0.296	−0.313	0.168	0.063
		Yes	0.039	0.034	0.252	−0.311	0.165	0.060
Pulsatility index	Crystallized	No	0.087	0.114	0.441	−0.617	0.349	0.077
		Yes	0.065	0.120	0.588	−2.280	0.857	0.008
	Memory	No	−0.006	0.167	0.971	−1.070	0.957	0.264
		Yes	−0.129	0.171	0.451	−0.675	1.151	0.558
	Speed	No	−0.650	0.150	0.000	0.119	0.339	0.726
		Yes	−0.598	0.155	0.000	0.293	0.357	0.411
	Visuospatial	No	−0.346	0.146	0.017	0.715	0.822	0.384
		Yes	−0.330	0.157	0.036	1.085	0.794	0.172
Resistivity index	Crystallized	No	0.324	0.388	0.404	−2.179	1.191	0.067
		Yes	0.244	0.411	0.553	−7.839	2.867	0.006
	Memory	No	0.079	0.569	0.889	−4.032	3.233	0.212
		Yes	−0.338	0.585	0.563	−2.745	3.916	0.483
	Speed	No	−2.222	0.512	0.000	0.769	1.140	0.500
		Yes	−2.026	0.529	0.000	1.353	1.199	0.259
	Visuospatial	No	−1.225	0.498	0.014	2.324	2.777	0.403
		Yes	−1.160	0.538	0.031	3.413	2.706	0.207

**Intercept and slope notes:**

Note: Carotid stenosis is the average of left and right ultrasound measures, in units of 10% of stenosis. Effects are shown for models without vascular risk factor adjustment (VRF) and models that include VRF as a time-varying covariate to indicate whether the stenosis effect is attenuated when VRF is controlled. Carotid velocity is the average of left and right, in units of 10% velocity measure. Carotid pulsatility is in units of the raw scale. Carotid resistivity is in units of the raw scale. The effect represents additive change in the intercept or slope per unit increase in stenosis, velocity, pulsatility or resistivity index.

After vascular risk factor adjustment, both pulsatility and resistivity indices were associated with the slope of decline in crystallised cognition between age 70 and 76 (estimate pulsatility − 2.280, *P* = 0.008; resistivity − 7.839, *P* = 0.006), but not with decline in processing speed, visuospatial function or memory, whether controlled for VRF or not (Table 4). The same pattern was seen when pulsatility and resistivity indices were analysed in standard deviation units (Supplementary Table 4).

## Discussion

Carotid stenosis has been proposed as a risk factor for cognitive decline and dementia. However, neither carotid stenosis nor ICA flow velocity explained the variance in the level of four key cognitive domains at age 70 in this highly characterised narrow-age cohort. Neither carotid feature was associated with the rate of decline in performance in any cognitive domain between ages 70 and 76 either, despite obvious declines in cognitive function over time in this key decade for cognitive ageing. Instead, measures of vascular stiffness, pulsatility and resistivity indices both associated with significantly poorer processing speed and visuospatial task performance at age 70, which persisted after correcting for age, premorbid IQ and vascular risk factors that reflected concurrent and historical disease. Hence, arterial stiffness rather than falling blood flow from cervical carotid obstruction contribute to impaired cognitive functioning among relatively healthy community-dwelling older adults. The presence of these associations at age 70, but general lack of association with decline in cognitive domains from age 70 to 76, suggests that the vascular stiffness-cognition association occurs over a very long period of time. This might indicate that midlife vascular disease is the trigger for cognitive decline in later life, or that some other as yet undetermined co-associated variable that increases vascular malfunction,^30^ is responsible.

The limitations include that few subjects had tight stenosis, but severe carotid stenosis is uncommon in community-dwelling subjects, or indeed even in patients with recent stroke where typically fewer than 10% have > 50% stenosis.^31^ However, we analysed the continuum of stenosis from none to severe on the basis that any effect of stenosis is unlikely to start only above a critical point. The follow-up period (six years) is relatively brief to detect effects on cognitive trajectories and longer periods should be examined. The cohort lacks ethnic diversity and the findings should be replicated in other populations. We cannot provide data on pulse wave velocity (not measured in LBC36) but PWV has been associated with cognition^32^ and WMH in other studies and we previously showed that PI and RI related to WMH in LBC36.^20^ Future studies should assess if PWV can substitute for carotid PI/RI measures as a risk predictor for failing cognition. We did not have carotid imaging at wave-1 or wave-3 but consider the wave-2 carotid data to be most relevant to our main interest which was the cognitive trajectory from age 70 to 76, rather than the intercept at age 70. We performed many comparisons, few of which were significant, and did not correct for multiple comparisons. However we modelled each cognitive domain separately; therefore, the number of directly competing comparisons is only a quarter of the total. Examination of adjusted and unadjusted model iterations, separate modelling of highly correlated pulsatility and resistivity, and two carotid stenosis measures also constituted a large degree of overlapping variance. We did not adjust for educational attainment, as do many studies of cognition in later life, because we had instead intelligence measured at age 11 (age 11 IQ). Age 11 IQ is closely related to educational attainment and to cognition at age 70+. We did not use education as well as age 11 IQ because the two are highly correlated and use of both would risk inflating the association with any variables of interest. We previously assessed the effects of education on cognition in later life after adjusting for age 11 IQ: education had an effect on some specific cognitive domains at age 70+ (the more verbal IQ tests) but not on processing speed, indicating that education may increase particular intellectual abilities^33^ but does not affect more fundamental capabilities like processing speed which underpin the efficiency of cognitive operations.^34^ The effect of education on these more verbal aspects of cognition is minor and therefore not justified in addition to age 11 IQ in this paper. Strengths include the narrow age cohort, availability of childhood intelligence, comprehensive measures of cognition in older age, large sample size, statistical power enabling reliable detection of small effects, and highly trained DUS operators.

To the best of our knowledge, no other studies have examined associations between four main carotid parameters and multiple domains of cognition during ageing, corrected for prior intelligence, used a narrow age cohort to minimise confounding by age, assessed longitudinal cognitive change, nor corrected for vascular risk factor exposure. Components of our results are supported by other studies.^9^ Review articles are inconclusive on whether carotid stenosis leads to cognitive decline.^9,35^ For example, declining cognitive function was associated with carotid plaque area but not luminal narrowing,^10^ or carotid volume flow.^36^ Removal of tight stenosis by carotid endarterectomy did not affect cognitive decline five years later.^15^ Carotid IMT, which was associated with vascular stiffening not stenosis, was associated with dementia,^11–13^ and low-ranked global cognitive function in middle-aged adults.^36^ Alzheimer’s disease is associated with reduced cerebral blood flow in cross-sectional analyses with age-matched controls but longitudinal data are lacking.^5^ Vascular stiffness (see Balucani in|^2^), rather than luminal narrowing, may be the relevant vascular problem. The increase in resistivity index points to intracranial vascular dysfunction, vessel stiffening, perhaps lack of reactivity to increased oxygen demand, or failed clearance of waste.^37^ WMH, which are associated with failing cognition also associate most strongly with increased vascular stiffness (of several carotid and systemic vascular measures) in numerous studies, including previous analysis from the LBC1936.^20^ In a small study in stroke-free subjects with hypertension, a wider CCA diameter and increased intracranial artery pulsatility index were associated with WMH.^38^ We also found no independent association between IMT^17^ or carotid stenosis^16,17^ and WMH in prior analysis in the LBC1936 or in patients with stroke.

The results may appear to disagree with experimental models of vascular dementia in which mild to severe carotid stenoses are induced by placing metal coils around the ICA.^7,39^ The affected rodents develop cognitive impairments and arteriolar changes resembling aspects of human lipohyalinosis. It is thought that the model works by reducing cerebral blood flow, but few studies measured CBF and placing coils on the carotid arteries may induce other effects like inflammation, reduced damping of the arterial waveform provided by the normal carotid bulb that protects the brain, or increased intracranial microvessel stiffness. Loss of normal damping of the arterial waveform would be consistent with our and other’s^40^ demonstration of increased carotid pulsatility and resistivity being associated with cognitive decline.

The slower rate of cognitive decline in subjects who were older at wave-1 requires some thought. Subjects who were older at wave-1 had poorer cognitive performance, and hence their rate of decline perhaps was slower because they had already declined compared with those who were younger at wave-1. Alternatively, this could reflect survivor bias: older subjects, who would be expected to have worse cognition at wave 1 versus younger subjects, even within this narrow age range, may represent the slightly healthier, slightly higher age 11 IQ end of their age group spectrum (non-participants either being to ill or not having survived), and therefore they declined more slowly than the younger wave-1 subjects amongst whom the health mix (and age11IQ) may have been wider with faster decline. Further longitudinal narrow age studies are needed to improve definition of cognitive trajectories stratified by baseline risk.

Vascular stiffening adversely influences cognitive ageing and provides a potential target for ameliorating cognitive decline. Future studies should assess several domains of cognition and carotid disease in diverse populations, ideally at younger ages, with long term follow-up, to determine when vascular stiffening starts. Carotid coil experimental models should assess flow velocities, pulsatility and resistivity indices to test if altered vascular stiffness, rather than flow reduction, might be responsible for the cognitive or pathological changes.

## Funding

The author(s) disclosed receipt of the following financial support for the research, authorship, and/or publication of this article: Funding from the Biotechnology and Biological Sciences Research Council (BBSRC) and Medical Research Council (MRC) is gratefully acknowledged. The authors’ institution received funding for the research from the Age UK Disconnected Mind Project, the Medical Research Council, The Chief Scientist Office of the Scottish Executive, and The Row Fogo Charitable Trust The Brain Research Imaging Centre, Edinburgh, receives funding from the Scottish Funding Council, the Chief Scientist Office and NHS Lothian Research and Development Office. The work was undertaken by The University of Edinburgh Centre for Cognitive Ageing and Cognitive Epidemiology, part of the cross council Lifelong Health and Wellbeing Initiative (MR/K026992/1).

## Supplementary Material

Supplementary material
