# Supplementary material for: Carotid disease at age 73 and cognitive change from age 70 to 76 years: A longitudinal cohort study
Source: J Cereb Blood Flow Metab. 2016 Jan 1;37(8):3042–52. doi: 10.1177/0271678X16683693 (PMC5536260; doi:10.1177/0271678X16683693)
Supplement: Supplementary material [file JCB683693_supplementary_material.pdf]

Supplemental Material

Cerebral vascular structure in the motor cortex of adult mice is stable and is not altered by voluntary exercise

Robert H. Cudmore[1], Sarah E. Dougherty[1] and David J. Linden[1*]

[1]Solomon H. Snyder Department of Neuroscience, The Johns Hopkins University School of Medicine, Baltimore, MD, USA
[*]To whom correspondence should be addressed: dlinden@jhmi.edu

Table of Contents

SUPPLEMENTAL METHODS **2**
Cranial window surgery. 2
In vivo two-photon microscopy 2
Immunohistochemistry 2
Vascular tracing 3
Longitudinal analysis of capillary segment diameter and length 3
Vascular dynamics 4
Network analysis 4
Statistical analysis 4
References 4

SUPPLEMENTAL FIGURES **5**
Supplemental Figure 1. Manual tracing. 5
Supplemental Figure 2. Distribution of capillary segment diameter and length. 7
Supplemental Figure 3. Visualizing smooth muscle and Virchow–Robin space surrounding surface and diving arteries. 8
Supplemental Figure 4. Fixed slice immunohistochemistry to identify pericytes 9
Supplemental Figure 5. Capillary segments bearing adherent pericyte cell bodies have reduced diameter. 10
Supplemental Figure 6. Addition of locked running wheel does not alter capillary segment diameter or length. 10
Supplemental Figure 7. Loops with immediate anastomosis are not pruned. 11

SUPPLEMENTAL TABLES **12**
Supplemental Table 1. Table listing all animals used in this study 12

SUPPLEMENTAL MOVIE LEGENDS **12**
Supplemental Movie 1. Movie of a 3D image volume 12
Supplemental Movie 2. Three-dimensional Y-Z projection of an image volume 12

Supplemental Methods

Cranial window surgery. To prepare cranial windows[1,2], Tie2-Cre:mTmG mice were anesthetized with isoflurane (1.5% mixed with O_2), and mounted in a stereotaxic frame. Body temperature was maintained at 37 °C with a feedback-controlled heating pad. A subcutaneous injection of dexamesthesone (0.05 ml at 2 mg/ml) was administered to reduce swelling and lidocaine (2%, 0.2 ml) was injected under the scalp to alleviate pain. The skull over the left cerebral hemisphere was exposed with a midline scalp incision and a 2 x 2 mm square region of skull over the motor cortex was removed using a #11 surgical blade. The medial-posterior corner of the craniotomy was located 0.5 mm lateral and 0.5 mm posterior to bregma which places the center of the craniotomy overlying the primary motor cortex.[3,4] Saline was applied throughout surgery to debride the tissue within the craniotomy. A 2 x 2 mm custom cut square coverglass (VWR, No. 1) was placed into the craniotomy so that it was flush with the skull and sealed using dental cement (C&B Metabond). Finally, a metal plate with a center hole was attached to the skull with dental cement to allow head fixation for two-photon imaging. All mice were individually housed following surgery. In vivo imaging began 2-3 weeks after cranial window implantation. Mice were excluded from the study if the cranial window was not suitable for sub-micron in vivo two-photon imaging.

In vivo two-photon microscopy. Mice were imaged using a two-photon microscope consisting of a Movable Objective Microscope (Sutter Instruments, Novato, CA) equipped with a MaiTai DeepSee laser (Spectra-Physics, Santa Clara, CA) tuned to 920 nm, a 20X, 1.0 N.A. water dipping objective (Zeiss), and GaAsP detectors (Hamamatsu, Japan). The microscope was controlled using ScanImage software (Version 3.8)[5] running in Matlab (Math Works).

Before each imaging session, mice were briefly (< 1 min) anesthetized with isoflurane and head fixed below the imaging objective. For awake imaging, mice were loosely restrained in a plastic tube, which allowed them to adjust their limb and trunk position but limited larger movements. For anesthetized imaging, 1.5% isoflurane in O_2 was delivered and body temperature was maintained with a feedback controlled heating pad. Imaging sessions typically lasted ~1 hour. All imaging was conducted in a dedicated microscope room during the light phase of the 12h/12h light dark cycle.

All images were acquired at a resolution of 1024 x 1024 pixels (0.216 µm/pixel, dwell time 1.6 µsec). Image stacks were acquired starting above the pial surface and continuing to depths of 400-500 µm with a step size of 2 µm. Vascular branch points were used to identify the same imaging area across sessions spanning many days. We were able to clearly image vascular segments in all image stacks (Supplemental Movies 1, 2).

To confirm that mGFP expressing vessels in Tie2-Cre:mTmG mice were patent, in a subset of mice we simultaneously imaged Texas Red-labeled dextran (70 kDa, Invitrogen D1830, tail vein injection) and found complete correspondence between mGFP expressing vessels and Texas Red filled vessels (images not shown).

Immunohistochemistry. Mice were anesthetized with intraperitonally injected ketamine (100mg/kg) and xylazine (10mg/kg) and then perfused with ice-cold phosphate buffered saline (PBS) followed by 4% paraformaldehyde in PBS. The entire brain was then dissected and kept in 4% paraformaldehyde overnight. The full brains were then cryoprotected with two subsequent overnight incubations in 15% and 30% sucrose in PBS at 4°C. The brains were sectioned on a sliding microtome (40 µm thick). Sections were washed in PBS then blocked with 5% normal goat serum (Jackson Immunoresearch #005-000-001, RRID: AB_2336983) and 0.3% TritonX100 in PBS at room temperature for 2 hr. Sections were then incubated in primary antibodies, diluted in 5% normal goat serum and 0.3% Triton-X100 in PBS, overnight at 4°C. The primary antibodies used were chicken anti-GFP (1:6000, Aves Labs #GFP-1010, RRID: AB_2307313) and rat anti-CD13 (1:500, Medical & Biological Laboratories Co., LTD. #M101-3, RRID: AB_590871). The sections were then washed in PBS and incubated in secondary antibodies at room temperature for

2 hr. The secondary antibodies used were Alexa Fluor 488-labeled goat anti-chicken (1:1000, Jackson ImmunoResearch #103-545-155, RRID: AB_2337390) and Cy5-labeled goat anti-rat (1:1000, Jackson ImmunoResearch #112-175-167, RRID: AB_2338264). The sections were then mounted on slides and motor cortex images were acquired using a single-photon confocal microscope (Zeiss) with a voxel size (in µm) 0.21 x 0.21 x 1.

Vascular tracing. Vascular structure was traced in 3D image stacks using the mGFP signal with a semi-automated workflow as follows. First, branch point positions were marked as 3D points and vessel segments between branch points were traced by manually specifying a number of intermediate 3D way-points (Supplemental Figure 1). Way-points followed the centerline of each vascular segment with the number of way-points per segment chosen by the scorer to match the local curvature of the segment. Thus, portions of each vessel segment with greater curvature had more way-points.

Vessel segment length was calculated as the sum of the straight-line 3D distance between 3D way-points for each segment. With this strategy, 3D vessel segment length calculations are reliable as subtracting a small number of way-points has little effect on the final 3D segment length (Supplemental Figure 1c-e). The largest source of variability in our 3D segment length measurement is small differences in the image plane where 3D branch points and 3D way-points are placed (± 1 image plane). Single image plane differences in the position of 3D branch points or 3D way-points were not adjusted across imaging sessions.

The diameter of each vessel segment is calculated as the average of the diameters at each 3D way-point belonging to that segment (Supplemental Figure 1f-g). To calculate the diameter at an individual way-point, a line was automatically drawn perpendicular to the axial direction of the vessel and the diameter of the vessel at the way-point was automatically calculated as the full-width-at-half-maximum of GFP intensities along this line (Mishra et al, 2014). The line intensity profile was taken from the single image plane occupied by the 3D way-point. Each automatic diameter calculation was visually verified and manually adjusted if it was in error. Errors in the automatic diameter measurements occurred in <5% of way-points and were usually due to GFP in circulating blood cells or GFP from another vessel near the vessel of interest.

Each vessel segment was manually annotated as either a pial vessel, a descending artery, an ascending venule, or a capillary segment. Capillary segments were defined as vessel segments within the brain parenchema and did not include pial vessels, descending arterioles, or ascending venules. Using this criterion gives an average capillary segment diameter of 5.03 µm ± 1.18 SD for 3476 capillary segments traced in 10 animals (Supplemental Fig 2). Capillary segments that were not fully contained within the image stack were not traced. This normally happened when one branch of a segment was visible but the other exited the image volume. The transition between neuronal layer I and II/III was marked where layer II/III cell bodies begin to appear using the red membrane-bound tdTomato signal and this transition was used to sort capillary segments into layer I and II/III categories.

To estimate the volume occupied by 3D branch points, we calculated the 3D convex hull enclosing annotated 3D branch points within individual image volumes using the Python SciPy library for QHull (http://www.qhull.org).

Longitudinal analysis of capillary segment diameter and length. Capillary segment diameter and length were longitudinally measured through multiple timepoints by having scorers, blind to the experimental condition, trace a pseudo-random subset of capillary segments (Fig 4 and Fig 5). This was done by tracing a subset of capillary segments in the first timepoint of interest and then independently tracing the same capillary segments in all other timepoints of interest. Scorers were instructed to trace at least 20 capillary segments evenly distributed through layers I and II/III. Using this procedure, the same subset of segments were traced in each timepoint within each animal.

Due to slight changes in the field of view from timepoint to timepoint, some vessel segments would enter or leave the imaging field of view across imaging sessions and so were not included in the analysis. The final number of capillary segments analyzed within each animal were constant (from time-point to time-point) but differed between animals due differences in the acquired image volumes between animals. During this tracing, we did not observe rapid dynamics such as vessel addition or subtraction occurring between sequential imaging sessions.

Vascular dynamics. To search for the addition and subtraction of capillary segments (Fig 6), two imaging sessions acquired ~3 months apart were selected for analysis from each longer time-series. All visible 3D branch points were annotated in each of the two imaging sessions (early and late). Then, the list of 3D branch points in the late session were visually iterated and matched with their corresponding branch point in the early session. During this branch point matching, all vessel segments emanating from each branch point were also matched. This 'backward' search was used to identify segment addition (segments present in the late session but not the early). This process was reversed and all 3D branch points in the early session were matched with their corresponding branch point and attached vessel segments in the late session. This forward search was used to identify segment subtraction (segments present in the early session but not the late). During this process we did not observe any vessel addition but did find occasional vessel subtractions (Fig 6). When a vessel subtraction was identified, intermediate time-points were searched to find when the subtraction began and completed. This strategy will find any persistently added and persistently subtracted capillary segments. To check if there were ongoing transient segment dynamics where segments were added and then subtracted (or subtracted to then be added again), we examined intermediate sessions in each timeseries and did not observe transient segments. Taken together, this strategy represents an exhaustive search of the entire imaging volume in each timeseries of each animal imaged.

Network analysis. To calculate vessel loop length (Fig 6c), the vascular tracing was converted to an undirected graph with branch points as nodes and vessel segments as edges. A depth-first-search algorithm was used to identify all loops from each branch point back to itself and find the shortest loop. Loop length is the number of vessel segments traversed to complete the loop. During this search, branch points that connect to vessel segments that were not scored (because they exited the imaging volume) were not traversed. Additional analysis was performed by including and excluding these boundary cases and did not change the results.

Statistical analysis. To compare all capillaries across all animals (group data in Figures 4c and 5c), we used GLMs with mixed effects to account for unequal sample sizes between animals and the repeated measurements for each individual capillary segment. GLMs were fit using the glmer() function from the lme4 package in R. A likelihood ratio test was used to generate p values by comparing a GLM including the factor of interest (e.g. early versus one month in Figure 4c and control, 24h, 48h, etc. etc. in Figure 5c) with a Null GLM with these factors removed.

References

1. Holtmaat A, Bonhoeffer T, Chow DK, et al. Long-term, high-resolution imaging in the mouse neocortex through a chronic cranial window. *Nat Protoc* 2009; 4: 1128–1144.
2. Zhang Y, Cudmore RH, Lin D-T, et al. Visualization of NMDA receptor–dependent AMPA receptor synaptic plasticity in vivo. *Nat Neurosci* 2015; 18: 402–7.
3. Paxinos G, Franklin KBJ. *The Mouse Brain in Stereotaxic Coordinates*. Gulf Professional Publishing, 2004.
4. Tennant KA, Adkins DL, Donlan NA, et al. The organization of the forelimb representation of the C57BL/6 mouse motor cortex as defined by intracortical microstimulation and cytoarchitecture. *Cereb Cortex* 2011; 21: 865–876.
5. Pologruto TA, Sabatini BL, Svoboda K. ScanImage: flexible software for operating laser scanning microscopes. *Biomed Eng Online* 2003; 2: 13.

Supplemental Figures

Supplemental Figure 1. Manual tracing.

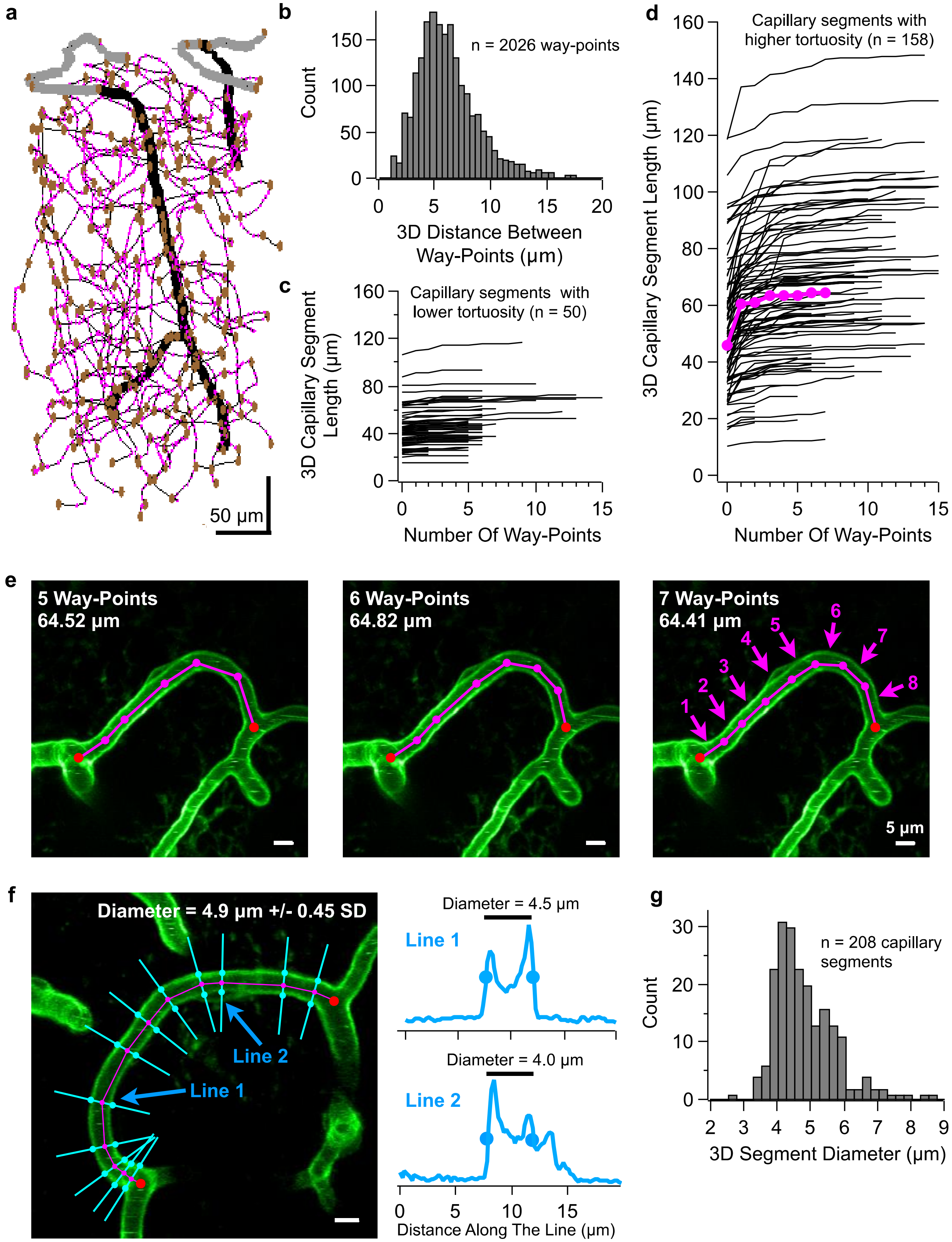

Supplemental Figure 1. Manual tracing.

(a) Representative vascular tracing for a 3D image volume. The pial surface is at the top of the panel and the volume extends ventrally for 504 µm (252 imaging planes) into the brain. Pial vessels are drawn in gray, diving arteries/veins are in black and vessel branch points are indicated by brown circles. The way-points are indicated as magenta circles, and the lines between way-points are drawn in black. Please note- capillary segment diameters are not represented here. In this example, there are 326 vascular branch points, 391 vascular segments (of which 208 are capillary segments), and 2617 way-points, of which 2026 are within capillary segments).

(b) A histogram of 3D distance between way-points for all capillary segments in panel (a). Capillary segments do not include pial and diving vessels. The average 3D distance between capillary segment way-points is 6.2 µm +/- 3.1 SD, n = 2026 way-points.

(c) Plot of 3D capillary segment length as a function of the number of way-points for capillary segments with lower tortuosity (n = 50 capillary segments). Each line represents the estimated length of one capillary segment as the number of way-points is increased. The number of way points used to calculate 3D segment length is the right-most point in each line. For visual clarity, we have arbitrarily separated the plots for straighter capillary segments (panel c) and more tortuous segments (panel d).

(d) Plot of 3D capillary segment length as a function of the number of way-points for capillary segments with higher tortuosity (n = 158 capillary segments). For each segment, the number of way-points used to calculate 3D segment length (the right-most point of each line) is on the asymptote where adding or subtracting individual way-points has little effect on the calculated segment length. One capillary segment with 7 way-points is highlighted in magenta and is shown in panel (e).

(e) One exemplar capillary segment showing three different tracings with three different numbers of way-points and the resulting estimate of 3D capillary segment length (5 way-points (left), 6 way-points (middle), and 7 way-points (right)). As the number of way-points varies by three, the calculated 3D segment length varies by < 1 µm. For a segment with a 3D length of ~64 µm, this represents a ~1% change. Branch points are red circles, way-points are magenta circles, magenta lines are 3D lines connecting way-point. 3D segment length is calculated by summing the 3D line length (magenta lines) between each way point and includes the 3D line length between terminal way-points and their corresponding branch point. Magenta numbers in right panel show the 8 3D line segments that are summed to obtain a 3D length of 64.41 µm. All branch points and way-points are manually specified using single image planes. For purposes of illustration, the images shown here are maximal Z-projections spanning ~20 µm in depth (10 image planes). Scale bar is 5 µm.

(f) One exemplar capillary segment showing individual diameter measurements at each way-point (12 way-points, 3D segment length of 84.2 µm). Blue lines are automatically drawn perpendicular to the direction of the traced segment and the image intensities (from the single image plane containing the way-point) along this line are used to automatically fit the diameter of the capillary segment at that way-point. Blue circles along each blue line denote the position of the diameter fit points. All automatic diameter measurements are manually verified and errors are corrected by manually adjusting the position of the fit points. Two example intensity profiles (Line 1 and Line 2) are noted in the image and shown on the right. The diameter of each capillary segment is the average diameter of all way-points in the segment, in this example it is 4.9 µm +/- 0.45 SD from n = 12 way points. All diameter measurements are performed on single image planes. In this example, there is a higher density of way-points in the lower portion of the image because the capillary segment began traveling in the z-direction, perpendicular to the imaging plane. For illustrative purposes, the image shown here is a maximal Z-projection of ~26 µm in depth (13 image planes). Scale bar is 5 µm.

(g) Histogram of 3D segment diameter for capillary segments (same tracing as in panel (a)). The average 3D segment diameter is 4.8 µm +/- 0.9 SD for n = 208 capillary segments.

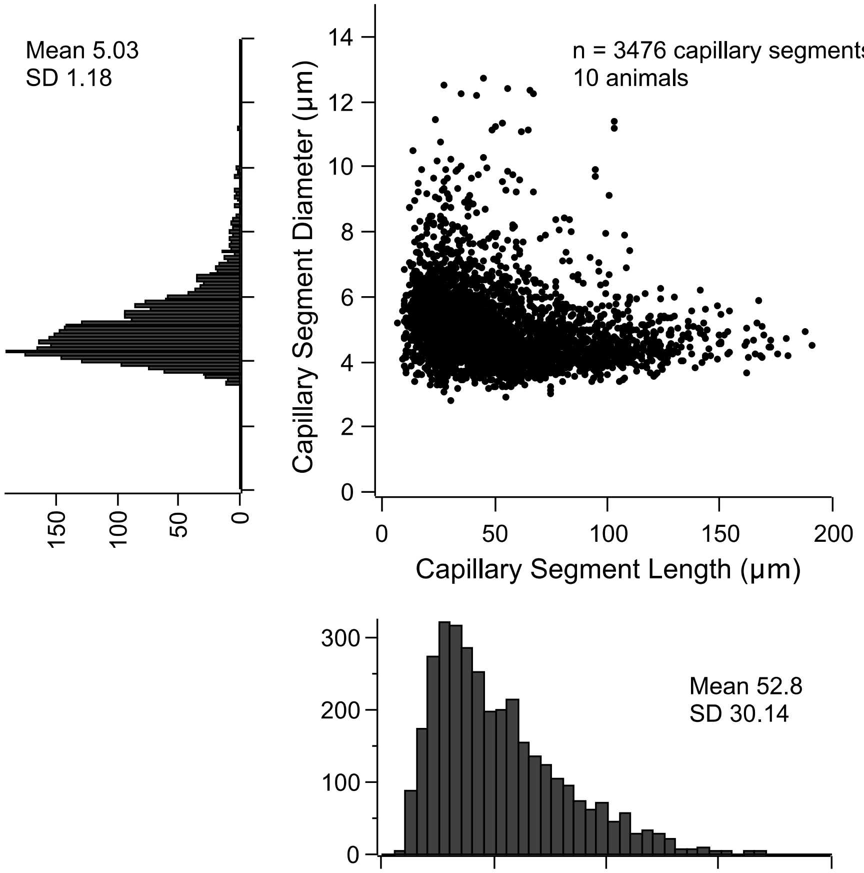

Supplemental Figure 2. Distribution of capillary segment diameter and length.

A scatter plot (top-right) of capillary segment diameter versus capillary segment length for 10 animals (n = 3476 capillary segments). Histograms show the distribution of capillary segment diameters (left) and length (bottom).

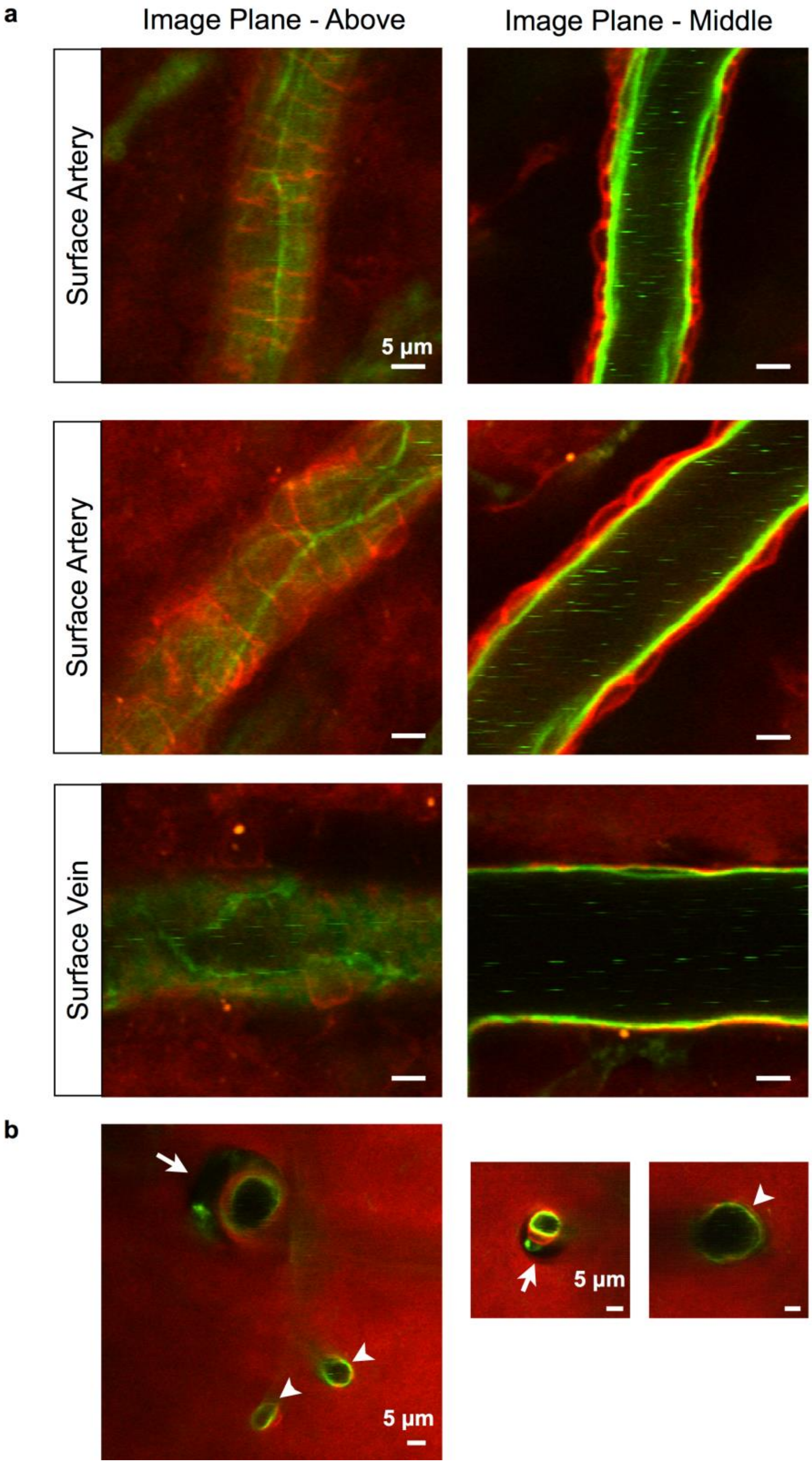

Supplemental Figure 3. Visualizing smooth muscle and Virchow–Robin space surrounding surface and diving arteries.

(a) Smooth muscle cells surrounding surface arteries appear as "belt-straps" of red fluorescence in imaging planes just above the endothelial wall of surface arteries (Surface Arteries, Image Plane - Above). Arterial smooth muscle cells are also visible in cross section when the imaging plane cuts through the middle of the surface artery (Surface Arteries, Image Plane - Middle). Smooth muscle cells are not present on surface veins (bottom row, Surface Vein).

(b) The Virchow-Robin space surrounding diving arteries is visible as a dark region (lacking membrane-bound Td-Tomato fluorescence) around diving arteries (arrows). Ascending venules do not have a visible Virchow-Robin space (arrow heads). Both large (left) and small (middle) diving arteries have a visible Virchow-Robin space. These images are from layer I, ~20 µm from the brain surface.

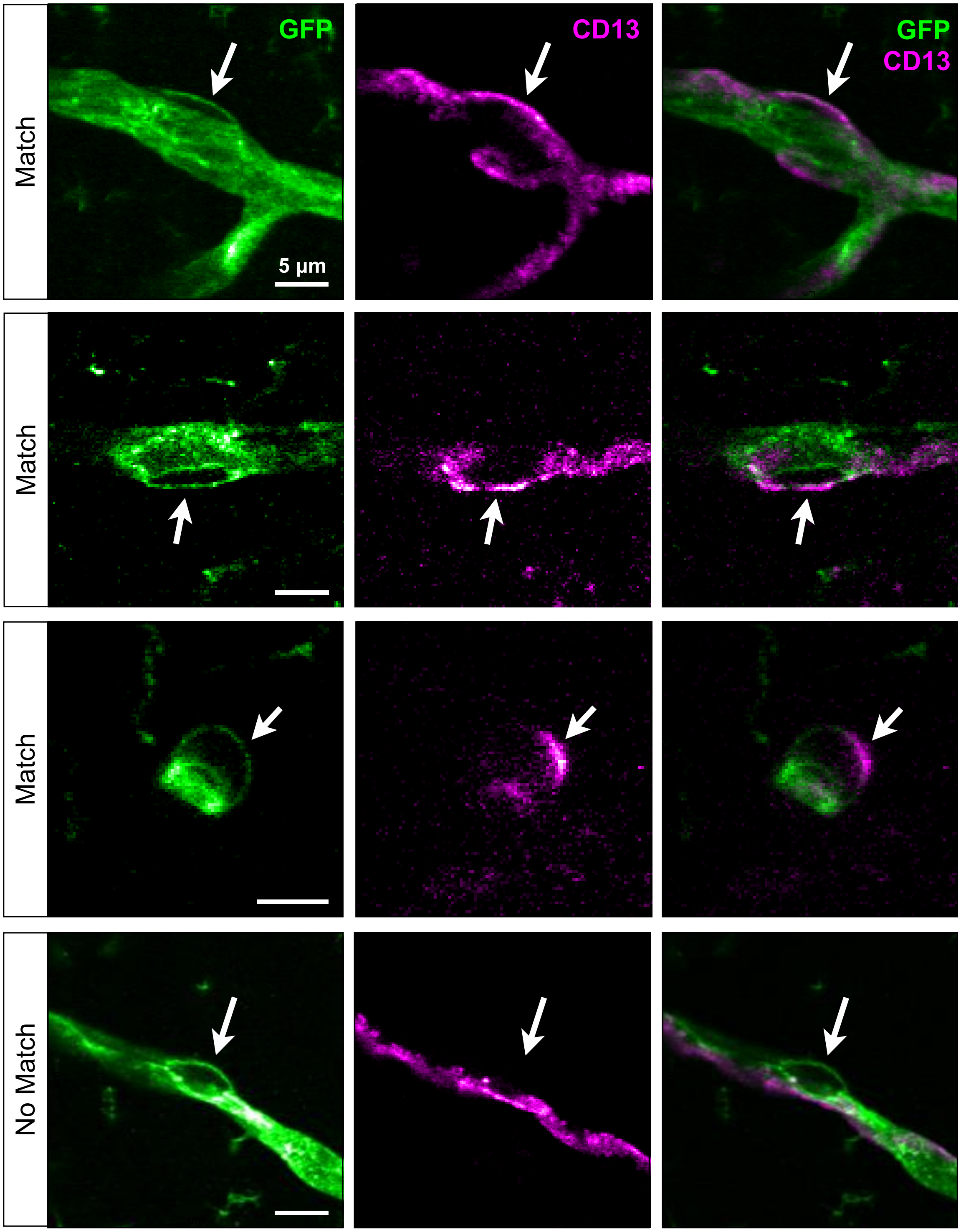

Supplemental Figure 4. Fixed slice immunohistochemistry to identify pericytes.
Exemplar images of double stained GFP (green) and CD13 (magenta) fixed tissue slices from Tie2-Cre:mTmG mice. Four example pericytes scored in the GFP channel (one per row) are shown to illustrate $GFP^+/CD13^+$ (top three rows) and $GFP^+/CD13^-$ (bottom row) cells. White arrows denote a pericyte that was scored using the GFP channel. The third row shows a $GFP^+/CD13^+$ pericyte associated with a capillary segment running perpendicular to the image plane and is an example where CD13 staining co-localizes with a portion of the membrane stained by GFP. Each image is a single confocal image plane and contrast has been adjusted for clarity. Scale bar is 5 µm.

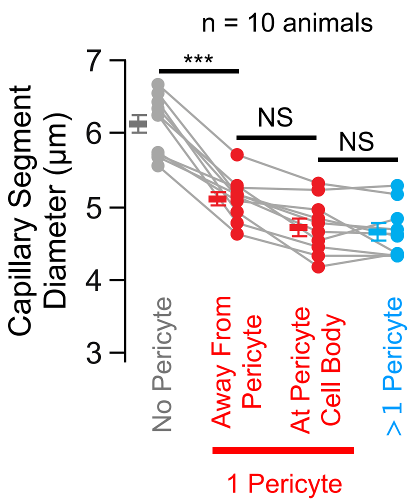

Supplemental Figure 5. Capillary segments bearing adherent pericyte cell bodies have reduced diameter.

Group plot of diameters for capillary segments with no pericytes (gray symbols, 506 segments), 1 pericyte cell body (red symbols, 421 segments), and more than one pericyte cell body (blue symbols, 136 segments). Gray lines connect measurements within the same animal (n = 10 mice). Capillary segments with one pericyte (red) have been grouped to show capillary segment diameter at the pericyte cell body versus a portion of the capillary at a distance from the pericyte cell body. There is no significant difference in capillary segment diameter away from versus at the pericyte cell body (n = 10 mice, ***$p < 0.001$, NS away versus at pericyte cell body $p = 0.32$, NS at pericyte cell body versus >1 pericyte $p = 0.67$, one-way ANOVA with Bonferroni post hoc test). This is an elaboration of the same data as is plotted in Figure 2, panel d, right side.

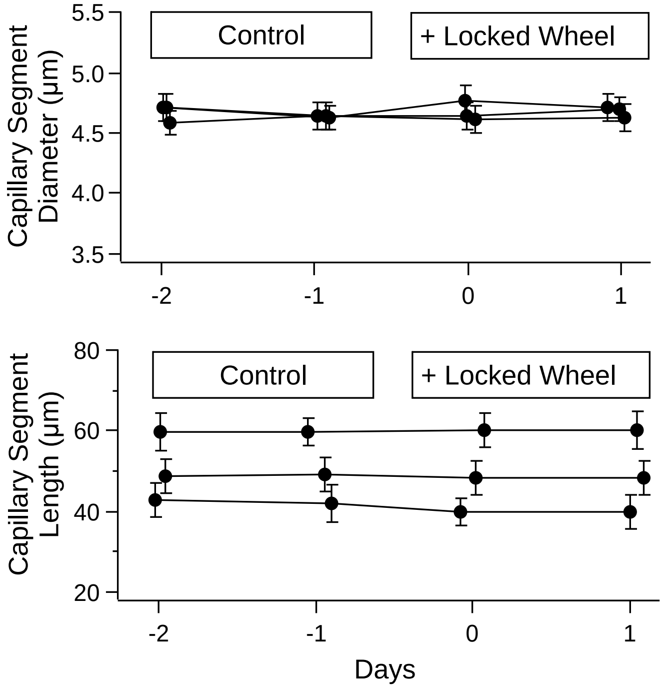

Supplemental Figure 6. Addition of locked running wheel does not alter capillary segment diameter or length.

Grouped data plots showing capillary segment diameter (top) and length (bottom) in control conditions and after the addition of a locked running wheel (+ Locked Wheel). Black symbols and lines connect measurements within individual animals (3 animals, diameter $p = 0.16$; length $p = 0.33$, GLM likelihood ratio test).

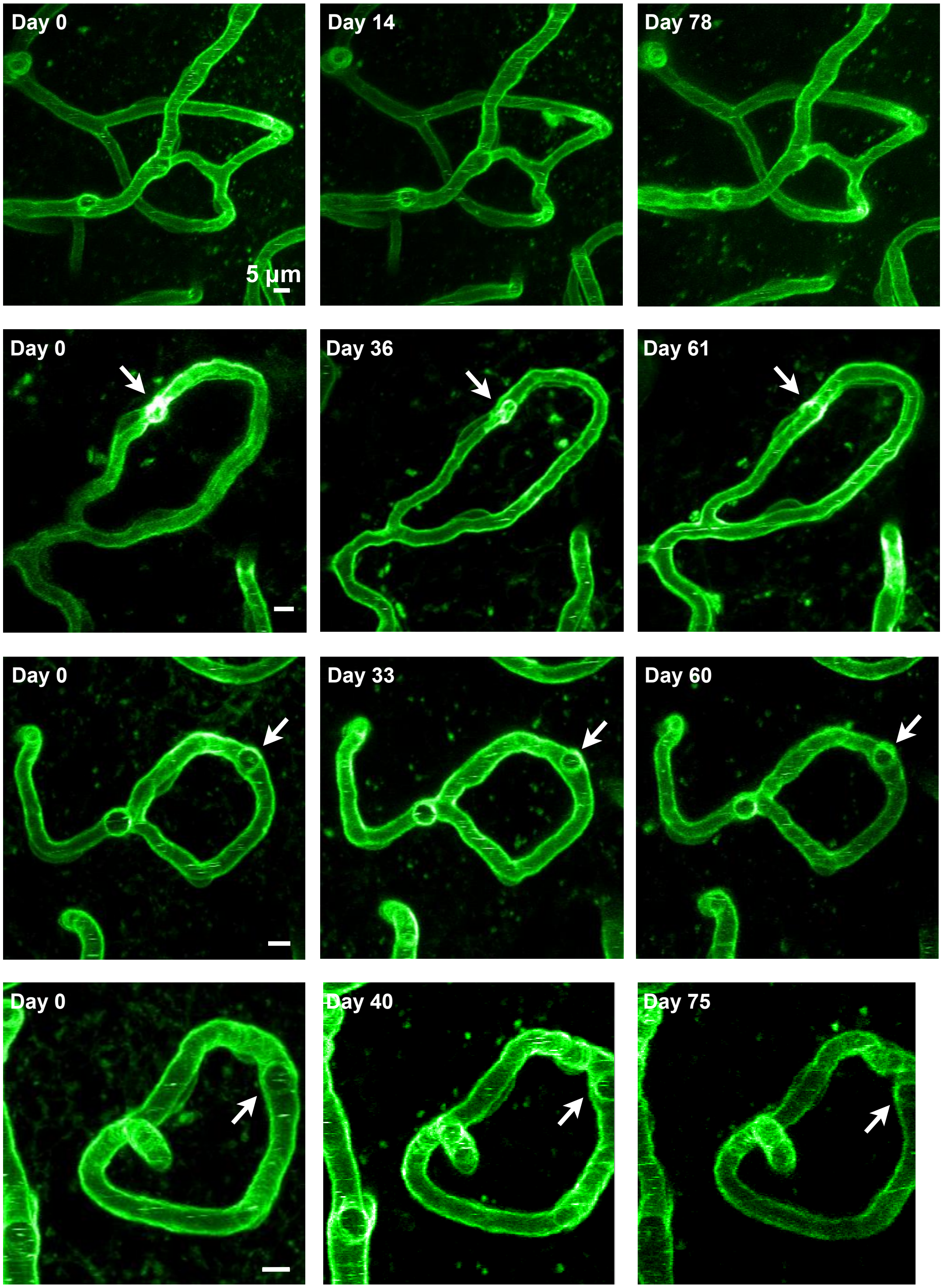

Supplemental Figure 7. Loops with immediate anastomosis are not pruned.
Exemplar time series of vascular loops with a length of two (immediate anastomosis). Each row shows a different vascular loop that is stable. Each image is a maximal Z-projection of a small number of single image planes chosen to show the topology of each loop. White arrows denote capillary segment branch points that give rise to an additional capillary segment that is not contained within the maximal Z-projection. Rows one, two and three are loops from three different animals. The loop depicted in row four is from the same animal as that shown in row three (n = 3 animals). Scale bars are 5 µm.

Supplemental tables

Supplemental Table 1. Table listing all animals used in this study. There are a total of 13 animals (one per row). Each animals' contribution to each data figure (Figures 1-7) is denoted with an 'x'. Postnatal day (pnd).

	**Animal ID**	**Surgery Age (pnd)**	**Surgery Age (months)**	**Sex**	**Days Run**	**Distance Run (km)**	**Figures**						
							**1**	**2**	**3**	**4**	**5**	**6**	**7**
1	a135	92	3.1	Male	38	146		x		x	x	x	
2	a136	94	3.1	Male	34	140		x				x	
3	a139	100	3.3	Female	None			x				x	
4	a141	96	3.2	Female	90	391		x		x	x	x	x
5	a144	449	15	Female	90	117		x		x		x	
6	a149	162	5.4	Female	None			x					
7	a152	681	22.7	Male	54	26		x			x	x	x
8	a153	135	4.5	Male	70	344	x	x		x	x	x	x
9	a154	139	4.6	Male	72	397					x	x	x
10	a156	100	3.3	Female	28	19			x	x	x	x	x
11	a157	102	3.4	Female	28	101			x	x	x	x	x
12	a164	161	5.4	Male	None			x	x	x		x	x
13	a165	162	5.4	Male	None			x	x	x			x

Supplemental movie legends

Supplemental Movie 1. Movie of a 3D image volume. Each frame in the movie corresponds to a single image plane. The depth from the pia is indicated in the upper right. The scale bar is 10 µm.

Supplemental Movie 2. Three-dimensional Y-Z projection of an image volume. This is the same volume shown in Supplemental Movie 1.
